# A Prospective Study of Usability and Workload of Electronic Medication Adherence Products by Older Adults, Caregivers, and Health Care Providers

**DOI:** 10.2196/18073

**Published:** 2020-06-02

**Authors:** Tejal Patel, Jessica Ivo, Sadaf Faisal, Aidan McDougall, Jillian Carducci, Sarah Pritchard, Feng Chang

**Affiliations:** 1 School of Pharmacy University of Waterloo Kitchener, ON Canada; 2 Centre for Family Medicine Family Health Team Kitchener, ON Canada; 3 Department of Family Medicine DeGroote School of Medicine McMaster University Hamilton, ON Canada; 4 Schlegel-University of Waterloo Research Institute of Aging University of Waterloo Waterloo, ON Canada

**Keywords:** electronic medication adherence, usability, workload, geriatrics, older adults, mobile phone

## Abstract

**Background:**

A decreased capacity to self-manage medications results in nonadherence, medication errors, and drug-related problems in older adults. Previous research identified 80 electronic medication adherence products available to assist patients with self-management of medications. Unfortunately, the usability and workload of these products are unknown.

**Objective:**

This study aimed to examine the usability and workload of a sample of electronic medication adherence products.

**Methods:**

In a prospective, mixed methods study, a sample of older adults, health care professionals, and caregivers tested the usability and workload of 21 electronic medication adherence products. Each participant tested 5 products, one at a time, after which they completed the system usability scale (SUS) and NASA-task load index (NASA-TLX), instruments that measure the usability and workload involved in using a product. Higher SUS scores indicate more user-friendliness, whereas lower NASA-TLX raw scores indicate less workload when using a product.

**Results:**

Electronic medication adherence products required a mean of 12.7 steps (range 5-20) for the appropriate use and took, on average, 15.19 min to complete the setup tasks (range 1-56). Participants were able to complete all steps without assistance 55.3% of the time (103 out of the 186 tests were completed by 39 participants; range 0%-100%). The mean SUS and NASA-TLX raw scores were 52.8 (SD 28.7; range 0-100) and 50.0 (SD 25.7; range 4.2-99.2), respectively, revealing significant variability among the electronic medication adherence products. The most user-friendly products were found to be TimerCap travel size (mean 78.67, SD 15.57; *P*=.03) and eNNOVEA Weekly Planner with Advanced Auto Reminder (mean 78.13, SD 14.13; *P*=.049) as compared with MedReady 1700 automated medication dispenser (mean 28.63, SD 21.24). Similarly, MedReady (72.92, SD 18.69) was found to be significantly more work intensive when compared with TimerCap (29.35, SD 20.35; *P*=.03), e-pill MedGlider home medication management system (28.43, SD 20.80; *P*=.02), and eNNOVEA (28.65, SD 14.97; *P*=.03). The e-pill MedTime Station automatic pill dispenser with tipper (71.77, SD 21.98) had significantly more workload than TimerCap (*P*=.04), MedGlider (*P*=.03), and eNNOVEA (*P*=.04).

**Conclusions:**

This study demonstrated that variability exists in the usability and workload of different electronic medication adherence products among older adults, caregivers, and clinicians. With few studies having investigated the usability and workload of electronic medication adherence products, no benchmarks exist to compare the usability and workload of these products. However, our study highlights the need to assess the usability and workload of different products marketed to assist with medication taking and provides guidance to clinicians regarding electronic medication adherence product recommendations for their patients. Future development of electronic medication adherence products should ensure that the target populations of patients are able to use these products adequately to improve medication management.

## Introduction

### Background

The global population is aging, and as a result, the proportion of older adults is growing rapidly. In 2017, there were 962 million individuals aged 60 years and older; by 2050, there will be 2.1 billion, accounting for 1 out of every 5 people [1]. With an increasing life expectancy, a greater proportion of the population is living well into their seventh and eighth decades, resulting in increases in the prevalence of chronic disease and comorbid medical conditions [2-4]. Indeed, in high-income countries, 86% of the burden of disease is due to noncommunicable chronic diseases; this is a phenomenon that is being replicated in middle- and low-income countries, as the ability to address infectious and parasitic diseases grows [2]. Chronic diseases such as heart disease, hypertension, diabetes, and cancer, among others, are typically managed with medications, in many cases, with multiple medications [5-7]. The use of multiple medications, commonly referred to as polypharmacy, for the treatment of comorbid conditions, brings its own set of complexities, including adverse effects, drug interactions, drug-induced disease, complex drug dosing regimens, and nonadherence, all of which increase the risk of hospitalization and mortality [5,8-10].

Medication nonadherence is a particularly problematic issue affecting older adults and results in suboptimal control of chronic conditions leading to poor health outcomes, hospitalizations, and significant health care costs [11,12]. For example, among patients aged 75 years and older, nonadherence to bisphosphonates resulted in an odds ratio of 1.49 for osteoporotic fractures and 13.4% higher medical costs than adherent patients [13]. Among older adults with epilepsy, the odds ratio of utilization of inpatient services was 0.66 among patients adherent to antiepileptic drugs compared with nonadherent patients and 13.2% less cost on the total direct health care costs [14]. Similarly, a population-level evaluation of the economic impact of nonadherence to medications used for the treatment of diabetes, heart failure, hyperlipidemia, and hypertension among Medicare beneficiaries in the United States demonstrated that improvement in adherence would result in an annual reduction of 117, 594 emergency department visits and over 7 million inpatient hospital days among patients with hypertension [15]. Likewise, reductions in these health care costs would also be realized in patients with hyperlipidemia, diabetes, and heart failure [15].

Adherence is defined as “the degree to which the person’s behavior corresponds with the agreed recommendations of a healthcare provider” [16]. Nonadherence to medications can arise from an intentional, active, and conscious decision to deviate, or result from passive, unintentional divergence from the prescribed dosing regimen [17,18]. Common causes of intentional nonadherence among older adults include beliefs related to the patient perception of illness and necessity for medications, experience of adverse events from medications, patient-prescriber relationship, and complexities related to dosing regimens of multiple medications [17]. Among older adults, nonadherence to medications can arise from several different factors, including patient-related, medication-related, health care provider–related, health care system–related, and socioeconomic-related factors [19]. Among the medication-related factors that can lead to inadvertent or unintentional nonadherence are dosing regimens, formulations, and packaging of medications [19]. Additionally, patient-related factors such as physical functioning (eg, vision limitations, impaired hearing, and poor dexterity) and cognitive impairment impact a patient’s ability to accurately administer medications, that is, the ability to take the right dose of the right medication at the right time [19-27]. These limitations are well recognized in both the clinical and research environments. As a result, several tools that measure the functional capacity of an older adult to manage medications have been developed to assist clinicians and patients in identifying different limitations in physical and cognitive capacities [28,29]. Several different strategies have been designed to address this need, including telephone reminders, memory aids and cues, pill boxes and dosettes, and compliance or blister-packaging, among others. However, systematic reviews indicate that the effectiveness of many of these strategies has produced mixed results in terms of the impact on adherence [30-32]. A systematic search for electronic products marketed to assist with medication management conducted in 2016 revealed that more than 80 such products were available for purchase to Canadians to address medication management needs [33]. These electronic medication adherence products may have audio and/or visual alarms, locking features, report-generating abilities, and real-time adherence monitoring, among others. These electronic medication adherence products are physical products that a patient can purchase and are not mobile or web-based apps. They may have a mobile or web-based app accessory to the product, but this is not the sole product. Very few of these commercially available electronic medication adherence products have been tested for usability and/or workload.

The usability of a product refers to the “facility with which users can use a technological artefact to achieve a particular goal” [34]. Before examining the effectiveness of these products on adherence and clinical outcomes, usability should be established. Products that are not user-friendly have the potential to worsen adherence in older adults. If a product is not practical, is socially unacceptable, has limited learnability, is inefficient and unpleasant to use, or results in increasing error rates, it may negatively affect adherence [35]. Usability is especially imperative to be established among older adults, given the higher prevalence of cognitive and physical impairments. A conceptual framework developed to address the usability of health technology among older adults highlights 4 different categories, including cognitive barriers, physical impairments, motivational issues, and perception barriers that impact usability and must be addressed [36]. However, usability of the majority of electronic medication adherence products has not been established. Of the products that have been tested, usability varies based on its features and limitations encountered in the participant population. In one study, 96 frail older adults used an electronic automated medication dispensing device for 1 year [37]. In this study, 94% (90/96) of the participants found the device to be easy to use, 95% (91/96) reported that it was reliable, and 95% (91/96) reported that the dispenser gave them peace of mind. However, only 84% (81/96) would consider using the dispenser in the future, and 5 participants indicated that they would require continued assistance from a nurse with pill setup. It is important to note that during this study, participants were not required to fill the dispenser with medications. In another study, 90% (18/20) of the 20 participants aged 55-75 years also indicated satisfaction with a personalized medication support system developed for Android platforms using quick response codes. However, users requested further improvements in printed characters, font sizes, and compatibility, and they noted financial constraints and the requirement of smartphones as limitations [38]. Similar limitations were identified in another study designed to investigate the usability of EMMA (R) among older adults [39]. Major challenges with usability were identified, including a narrow medication loading slot; difficulty in reading the font; and difficulty in identifying, retrieving, and opening delivered medications. In a separate study, usability of medication adherence technology was found to be limited in persons with cognitive impairment [40]. In this study, Mini-Mental Status Examination (MMSE) scores were significantly correlated with the percentage of task success; noncognitively impaired individuals completed 69% of the tasks required to use an integrated medication unit, whereas cognitively impaired individuals were only successful at completing 34.7% of the tasks [40]. Similar issues with navigation, poor visibility, and lack of transparency have been identified in medication management apps available for use in smartphones [41,42]. Usability scores of some of these apps ranged from 42 to 57 on the system usability score (SUS), a validated measure of usability where products are scored from 0 to 100, with higher scores indicating better usability [41,42].

### Objectives

Given that these studies demonstrated significant variability and limitations in the usability of health technology addressing medication management capacity and adherence and that the usability of many commercially available electronic medication adherence products has not been tested, this study aimed to examine the usability and workload of a range of electronic medication adherence products among older adults, caregivers, and health care providers.

## Methods

### Study Design

This study was designed as a prospective, mixed methods study to investigate the usability and workload of 21 electronic medication adherence products. In an effort to measure usability in a multidomain approach, we used both quantitative measures, including validated tools, cognitive walkthroughs, observations, and qualitative one-on-one interviews [35,43]. The paper describes the quantitative findings of this study. We did not develop any of the products tested in our study.

### Sample and Sample Size

We used purposive sampling techniques to recruit three types of participants for this study: older adults, caregivers of older adults, and health care providers. To be eligible, older adults had to be aged 65 years and older and taking one or more prescription or nonprescription medications regularly, whereas caregivers had to be assisting older adults with medication administration. Older adults and caregivers were recruited through advertising, professional networks, and from a participant pool of older adults who had previously indicated an interest in participating in research. All health care providers were eligible for the study and were recruited primarily through professional networks of researchers.

As 80% of the usability problems can be identified with 5 test-users [34], we aimed to test each product with at least five participants. Therefore, we targeted a recruitment sample of 25, composed of 15 older adults or caregivers and 10 health care providers.

### Products Included

In total, 23 electronic medication adherence products were purchased for the purposes of this study; however, 1 product was nonfunctional and, as such, was not tested. One product was found to be nonelectronic; however, it was still tested, although the results are not included in this report. Products were chosen with the objective of representing different features such as automated dispensing, number and type of compartments, audio and vibration reminder alarms, cloud connectivity, and medication dispensing tracking. Products that cost over Can $1000 (US $710.84) to purchase were not selected based on funding constraints. The names and details of the electronic medication adherence products tested are described in Table 1.

**Table 1:** Electronic medication adherence products tested.

Name of product	Abbreviated name	Manufacturer	Price (US $)
GMS^a^ Med-e-lert automatic pill dispenser^b^	Med-e-lert	Group Medical Supply, LLC^c^	70-109
LiveFine automatic pill dispenser and reminder ^b^	LiveFine	LiveFine	70-109
MedReady 1700 automated medication dispenser^b^	MedReady	MedReady Inc	≥109
MedSmart med-reminder and dispensing system	MedSmart	e-pill^d^	≥109
e-pill MedTime Station automatic pill dispenser with tipper	MedTime Station	e-pill	≥109
TimerCap travel size	TimerCap T	TimerCap, LLC	<30
TimerCap universal size	TimerCap U	TimerCap, LLC	<30
Jones medication adherence system 14-unit card	Jones	Jones Packaging Inc	N/A
Reizen vibrating pill box	Reizen	Reizen	<30
VitaCarry advanced pill case^b^	VitaCarry	VitaCarry	30-69
Nishiki round pill box with alarm^b^	Nishiki	Nishiki	<30
MedGlider system 1 with talking reminder^b^	Medport MedGlider	Medport	30-69
Patterson medical tabtime super 8^e^	TabTime	Tabtime LTD^f^	30-69
100-Hour pill reminder^e^	100-Hour	Aidapt	<30
Med-Q smart pill box	Med-Q	Med-Q	70-109
e-pill MedGlider home medication management system	e-pill MedGlider	e-pill	30 - 69
MedCentre system^b^	MedCentre	MedCenter Systems, LLC	30-69
eNNOVEA Weekly Planner With Advanced Auto Reminder	eNNOVEA	eNNOVEA Medical, LLC	70-109
e-pill Multi-alarm pocket XL	Multi-Alarm	e-pill	30-69
6 Grid pill storage case with alarm	Pill Storage Case	NR	<30
Itzbeen pocket doctor^b^	Pocket Doctor	Itzbeen	<30

^a^GMS: Group Medical Supply.

^b^Purchased from Amazon Canada or the United States.

^c^LLC: Limited Liability Company.

^d^e-pill: electronic pill.

^e^Purchased from Ebay Canada.

^f^LTD: Limited.

### Outcome Measures

#### Usability: System Usability Scale

SUS is a popular end-of-test subjective assessment of the usability of a product [44,45]. It is a validated, quick, and easily administered tool that has been used to test products across a wide range of industries. It consists of 10 statements (5 positive and 5 negative), which are scored immediately after testing a product on a 5-point Likert scale. Scores range from 0 to 100, with higher scores indicating that a product is more usable.

#### Workload: NASA-Task Load Index

The NASA-task load index (NASA-TLX) measures the workload required to complete a task [46]. It consists of 6 subscales: mental, physical, demand, frustration, effort, and performance. Participants were asked to rate each of the above-mentioned variables on a 20-point scale that measures from high to low (scored from 0 to 100) [46]. The tool has been applied in various fields, such as measuring workload in persons working in critical care, performing surgery, commercial aviation, and daily activities (such as operating a medical device at home) [47]. There are 2 ways to calculate the score. For the purpose of this study, we calculated the average of the ratings of the 6 items for each participant [47]. Lower scores indicate less workload.

#### Task Completion Times, Task Completion Rates, and Error Rates: Cognitive Walkthrough Checklist, Thinking Aloud

A cognitive walkthrough checklist was used to portray the cognitive tasks an individual performs while completing a series of complex tasks [48]. Individual cognitive walkthrough checklists were developed for each tested electronic medication adherence product. Cognitive walkthroughs were designed as outlined by Kushrinuk and Patel [48] and included (1) identifying the end users of the medication adherence products (older adults, caregivers, and health care providers), (2) defining the tasks for the walkthrough based on instructions provided by the manufacturer and supplemental information available on the web, and (3) walking through the actions and critiquing the vital information and steps with several pilot runs by research team members. Any critical steps, defined as those that were necessary for the appropriate use of the product, were identified and noted. The cognitive walkthrough checklist for each product listed each task, the steps required to complete the task, space to note errors performed in completing the tasks as well as time to complete, and if the completion of all the tasks was unassisted or partially assisted. The tasks varied for each product, but generally included steps such as opening and filling a tray with medications by using a mock medication regimen with placebo tablets and capsules, setting an alarm, locking a device, and removing the placebo tablets or capsules. As participants tested each product, they were invited to *think aloud*, that is, verbalize their thoughts as they completed the tasks required to use a medication adherence product with the mock medication regimen [48]. The *think aloud* sessions were audio recorded using a digital recorder (Sony IC Recorder ICD-PX470), while also being observed by a research team member who recorded their observations on the cognitive walkthrough checklist. Having the patient verbalize their thoughts helped researchers gain insight into what the participant was thinking and why certain errors were occurring. Participants were timed from the point at which they started testing the adherence product up to the point at which they completed all the tasks required to use the product or the point at which the participant refused to continue because of frustration, confusion, or fatigue with completing the remaining tasks. Timing was measured using an Apple iPhone X (software version iOS 13.31, 2017) clock app with a built-in timer.

#### Mock Medication Regimen

A mock polypharmacy medication regimen was developed for the purpose of this study. Placebo tablets (national product number 00501190, Odan Laboratory, lot #188628A, 100 mg lactose tablets), candy (Tic Tac), and placebo capsules (manufactured in-house) were used to represent the following medications (with administration instructions as indicated here): warfarin 2 mg once daily on Monday, Wednesday, and Friday and 3 mg once daily on Tuesday, Thursday, Saturday, and Sunday; pantoprazole 20 mg twice daily; phenytoin 100 mg, 1 capsule in the morning and 2 capsules in the evening; and propranolol 20 mg, half a tablet once daily for 2 days, then 1 tablet daily. The dosing regimen was designed to reflect commonly prescribed regimens. Participants were required to use the mock medications and instructions provided on the prescription labels to fill the medication adherence products they were testing in an effort to reflect activities conducted in real-life scenarios. For each product participants tested, they were required to use the same mock regimen.

#### Testing Procedures

Each participant tested 5 electronic medication adherence products. No training was provided to participants as this option would not be available in the community setting, but participants were provided with manufacturer instructions. If manufacturer instructions were not enclosed with the product, researchers accessed web-based instructions and provided them to the participant. Each participant was required to perform a series of tasks while *thinking aloud*, covering all aspects of using the product. At the end of each product testing, participants were asked to complete both the SUS and NASA-TLX. Finally, each participant was invited to an optional one-to-one semistructured interview examining participant perceptions about the features of the products, if they would consider using the product for managing their medications or if they would recommend the product to friends or family members. The results of the qualitative analysis for the one-to-one interviews are not presented in this analysis.

### Data Collection and Statistical Analysis

The SUS and NASA-TLX were provided to participants to complete in a paper format at the end of each product testing. The cognitive walkthrough checklist was completed by a research team member for each product. The audio recordings were then used to ensure the accuracy of the observations completed by a second team member. Data were entered in Microsoft Excel spreadsheets (Microsoft Excel version 16.16.14, 2016), Microsoft Word (Microsoft Word version 16.16.13, 2016), and Microsoft Access (Microsoft Access Version 16.0.4738.100, 2016).

SUS and NASA-TLX scores for the different electronic medication adherence products were analyzed using RStudio version 3.5.1 (2018-07-02). A repeated-measures analysis of variance (ANOVA) was used to compare both SUS and NASA-TLX scores of all the products, followed by Tukey posthoc analysis. The Pearson correlation was used to determine if there was a statistically significant relationship between SUS scores, that is, usability and NASA-TLX scores, that is, workload.

Error rate was calculated by dividing the number of total errors made (per person per product) by the total number of steps required to use the product (per product). Mean error rates are reported as percentages. Unassisted task completion rates were calculated by dividing the total number of tasks completed without assistance by each participant for each product by the total number of tasks required to use the particular product. The means of this measure are provided as percentages. Completion rates were calculated as the total number of tasks completed with or without assistance divided by the total number of tasks. Completion times reported are the mean time required to complete the setup and use of each product per participant. Error rates, completion times, and completion rates for each electronic medication adherence product tested are summarized.

### Ethical Review and Location

This study was reviewed by and received approval from the University of Waterloo Office of Research Ethics. All participants were informed of the study and provided consent before enrolling. This study was conducted at the University of Waterloo School of Pharmacy in Kitchener, Ontario, Canada.

## Results

### Participant Demographics

A sample of 39 individuals were recruited to test the electronic medication adherence products in this study, of which 23 were older adults, 5 were caregivers, and 11 were health care professionals. The majority of the 23 older adults were taking more than 5 medications, including prescriptions, vitamin supplements, over the counter drugs, and/or natural health products concurrently (see Table 2).

Almost 70% (16/23) of older adults also reported using a medication aid to assist with medication management, with the most commonly utilized being pill boxes, followed by alarms on a phone/watch and blister pack. All 5 caregivers assisted older adults with their medication management. Of the 11 health care providers, all of them worked with older adults in some capacity, with more than 80% (9/11) assisting with medication taking activities, but all recommending some type of medication aid to older adults (see Table 3).

**Table 2:** Demographic characteristics of older adults and caregivers.

Variable			Older adults (n=23)	Caregivers(n=5)
**Gender, n (%)**				
	Male		11 (48)	4 (80)^a^
	Female		12 (52)	1 (20)^a^
**Age (years)**				
	Mean (SD)		75 (6.7)	73.2 (4.49)^a^
	Mode		82	N/A^b^
	Median		75	71^a^
	Range		65-87	69-79^a^
**Number of medications taken per participant**				
	Mean (SD)		7.5 (3.3)	9.8 (5.3)^a^
	Mode		8	N/A
	Median		8	8.5^a^
	Range		1-13	5-17^a^
	Total number of drugs		172	39^a^
**Number of participants taking more than 5 medications per day, n (%)**				
	Prescription medications, OTC^c^, vitamin supplements, and herbal		19 (83)	—^d^
	Prescription medications		10 (44)	—
	OTC, vitamin supplements, and herbal		6 (26)	—
**Medication schedule, n (%)**				
	Once daily		4 (17)	0 (0)^a^
	More than once daily		19 (83)	5 (100)^a^
**Medication aids use, n (%)**				
	**Yes**			
		Total	16 (70)	5 (100)^a^
		Blister pack	3 (13)	2 (40)^a^
		Pill box	12 (52)^e^	4 (80)^a^
		Alarm (phone and/or watch)	4 (17)	0 (20)^a^
		App	1 (4)	0 (20)^a^
		Calendar and/or message board and/or list	2 (9)	1(20)^a^
	No		7 (30)	0 (0)^a^
**Medication aids used in combination, n (%)**				
	**Yes**			
		Total	5 (22)	2 (40)^a^
		Pill box and alarm	2 (9)	0 (0)^a^
		Pill box and message board and/or list	2 (9)	1 (20)^a^
		Pill box and app	1 (4)	0 (0)^a^
		Pill box and blister pack	0 (0)	1 (20)^a^

^a^Reported by caregiver for the patient.

^b^N/A: not applicable.

^c^OTC: Over the counter.

^d^Not reported.

^e^One participant reported not using any medication aid but stated that they used a pill case only for traveling purposes.

**Table 3:** Demographic characteristics of health care providers.

Variable			Health care providers (n=11)
**Gender, n (%)**			
	Male		2 (18)
	Female		9 (82)
**Occupation, n (%)**			
	Pharmacist		8 (72)
	Pharmacy student		1 (9)
	Occupational therapist		2 (18)
**Years of practice**			
	Mean (SD)		8.8 (10.5)
	Mode		1
	Median		5
	Range		0^a^-37
**Older adults worked with/dispensed prescriptions for, n (%)**			
	<10		1 (9)
	10-20		4 (36)
	20-30		1 (9)
	>30		5 (46)
**Assist older adults with medication taking, n (%)**			
	Yes		9 (82)
	No		2 (18)
**Medication aids recommendation, n (%)**			
	**Yes**		
		Total	11 (100)
		Blister pack	10 (91)
		Pill box/dosette	6 (55)
		Easy snap cap	1 (9)
		Alarm	3 (27)
		Phone app	1 (9)
	No		0 (0)

^a^One health care provider was a pharmacy student and thus had 0 years of practice as a registered pharmacist.

### Usability

The overall mean SUS score for all 21 products tested was 52.28 (SD 28.52; range 0-100). The mean SUS score per product ranged from a low of 28.63 (SD 21.240) for the MedReady 1700 automated medication dispenser to a high of 78.67 (SD 15.572) for the TimerCap travel size (Table 4). The mean SUS scores were significantly different between MedReady 1700 automated medication dispenser and the TimerCap travel size (mean 28.63, SD 21.24 vs mean 78.67, SD 15.57; *P=*.03) and MedReady 1700 automated medication dispenser and the eNNOVEA Weekly Planner with Advanced Auto Reminder (mean 28.63, SD 21.24 vs mean 78.13, SD 14.13; *P=*.049).

**Table 4:** Mean system usability scale and NASA-task load index scores

Product name	System usability scale		NASA-task load index	
	Score, mean (SD)	Range	Score, mean (SD)	Range
Med-e-lert	40.75 (31.78)	7–85	67.08 (25.60)	33.33–97.50
LiveFine	46.50 (27.38)	10–85	65.21 (21.32)	32.50–99.17
MedReady	28.63 (21.24)	0–65	72.92 (18.69)	43.33–92.50
MedSmart	41.44 (33.97)	5–87	59.23 (25.82)	13.33–93.33
MedTime Station	35.13 (17.84)	20–72	71.77 (21.98)	2.67–99.17
TimerCap T	78.67 (15.57)	50–100	29.35 (20.35)	4.17–55.83
TimerCap U	56.11 (30.92)	0–92	40.56 (27.72)	12.5–99.17
Jones	50.64 (24.63)	7–92	48.63 (18.11)	23.33–85.83
Reizen	50.67 (24.06)	20–92	50.28 (27.46)	4.17–76.67
VitaCarry	52.78 (31.69)	0–87	46.11 (23.45)	13.33–83.33
Nishiki	46.89 (26.50)	0–90	56.48 (26.12)	11.67–97.5
Medport MedGlider	57.20 (30.85)	0–95	45.75 (24.05)	18.33–95.00
TabTime	57.89 (31.36)	0–97	35.65 (20.30)	4.17–68.33
100-Hour	55.78 (25.28)	2–85	49.35 (24.57)	26.67–95.83
Med-Q	64.60 (27.85)	12–90	42.17 (28.70)	14.17–89.17
e-pill MedGlider	71.67 (30.84)	5–100	28.43 (20.80)	4.17–69.17
MedCentre	56.63 (22.10)	37–92	52.19 (18.52)	29.17–79.17
eNNOVEA	78.13 (14.13)	55–97	28.65 (14.97)	10.00–57.5
Multi-Alarm	51.56 (24.59)	15–95	45.65 (25.79)	4.17–78.33
Pill Storage Case	34.75 (22.60)	0–62	65.10 (14.17)	41.67–80.83
Pocket Doctor	35.00 (33.19)	0–92	68.33 (27.37)	20.00–93.33

### Workload

For the 21 products tested, the overall mean NASA-TLX score was 50.43 (SD 25.49; range 4.17-99.17). The mean NASA-TLX score per product ranged from 28.65 (SD 14.97) for the eNNOVEA Weekly Planner with Advanced Auto Reminder to 72.92 (SD 18.69) for the MedReady 1700 automated medication dispenser (Table 4). Similar to SUS scores, ANOVA analysis revealed significant differences in the NASA-TLX raw scores. The NASA-TLX raw scores for the MedReady 1700 automated medication dispenser (mean 72.92, SD 18.69) were significantly higher than those for the TimerCap travel size (mean 29.35, SD 20.35; *P=*.03), e-pill MedGlider home medication management system (mean 28.43, SD 20.80; *P=*.02), and eNNOVEA (mean 28.65, SD 14.97; *P=*.03). The e-pill MedTime Station automatic pill dispenser with tipper (mean 71.77, SD 21.98) demonstrated significantly higher workload than TimerCap (*P=*.04), MedGlider (*P=*.03), and eNNOVEA (*P=*.04).

### Correlation Between System Usability Scale Scores and NASA-Task Load Index Scores

The SUS scores were highly correlated with NASA-TLX scores (Pearson *r*=−0.877; 95% CI −0.907 to 0.839; *df*=184; *P*<.001), such that an increase in usability of the product was inversely correlated with decreasing workload involved in using the product.

### Mean Time to Task Completion, Task Completion, and Error Rates

The number of tasks and the number of steps within each task to use a product varied depending on the characteristics and features of the products. Products required an average of 12.7 steps (SD 4.1; range 5-20) for use and included steps such as inserting the key into the lock, rotating the key clockwise, and pinching the tab to open the product. These steps were grouped into tasks such as opening the product, loading the product with placebo pills or Tic Tac candy as per the standardized mock medication schedule provided, closing the product, setting an alarm, and removing the medications from the product after the alarm sounds (Table 5). On average, with assistance from a research team member, participants were able to complete 97% of the tasks required to use a product (SD 8.97%; range 16.7%-100%). However, without the assistance of a researcher, participants were only able to complete 100% of all steps required to use a product in 103 of 186 product tests (103/168, 55.3%; range: 0%-100%).

**Table 5:** Mean error rates, completion time, unassisted task completion, and completion rates of electronic medication adherence products tested.

Abbreviated product name	Total number of steps	Description of tasks					Mean time to task completion (min:sec)		Mean error rate (%)		Mean unassisted task completion (%)		Mean completion rate (%)	
		Open and fill medication tray	Set alarm	Lock device	Remove medication from device	Other								
Med-e-lert	18	X^a^	X	X	X	N/A^b^		17:24		22		81		98
LiveFine	18	X	X	X	X	N/A		18:22		17		70		90
MedReady	16	X	X	X	X	N/A		26:15		21		86		98
MedSmart	20	X	X	X	X	N/A		25:20		19		67		98
MedTime Station	17	X	X	N/A	X	X		31:13		36		66		98
TimerCap T	5	X	X	N/A	N/A	X		5:36		2		100		100
TimerCap U	5	X	X	N/A	N/A	X		5:19		7		100		100
Jones	15^c^	N/A	N/A	N/A	X	X		15:17		35		80		92
Reizen	10	X	X	N/A	X	N/A		15:29		15		78		95
VitaCarry	10	X	X	N/A	X	N/A		15:11		20		88		99
Nishiki	10	X	X	N/A	X	N/A		15:10		26		82		98
Medport MedGlider	11	X	X	N/A	X	N/A		16:16		10		95		100
TabTime	12	X	X	N/A	X	N/A		12:20		16		81		99
100-Hour	10	X	X	N/A	X	N/A		9:16		3		96		100
Med-Q	12	X	X	N/A	X	N/A		12:16		27		85		100
e-pill MedGlider	14	X	X	N/A	X	N/A		10:13		17		90		100
MedCentre	15	X	X	N/A	X	N/A		16:23		10		72		94
eNNOVEA	14	X	X	N/A	X	X		15:31		10		88		98
Multi-Alarm	12	X	X	N/A	X	N/A		11:22		25		78		97
Pill Storage Case	12	X	X	N/A	X	N/A		15:32		24		79		95
Pocket Doctor	17	N/A	X	N/A	X	X		15:11		24		68		85

^a^Task needed to be completed.

^b^N/A: not applicable.

^c^A total of 3 tasks (of the total 15) were only applicable for health care providers to complete. Thus, the total number of steps is 12 for an older adult or caregiver participant, and it is 15 for a health care provider participant.

## Discussion

### Principal Findings

To our knowledge, our study is the first and the largest study to examine and compare the usability and workload of electronic medication products in a population of older adults, caregivers, and health care providers. We tested a range of products, varying between products with few features to complex devices with multiple features, using a standardized mock medication regimen that mimicked polypharmacy and medication regimen complexity that is prevalent in an older adult population [5]. The electronic medication adherence products we purchased were discovered in a systematic search we conducted in a previous study and reflected a variety of characteristics and available features [33]. The results of our study demonstrate significant variability in both the usability and workload required to use electronic medication adherence products. Additionally, the task completion rates varied between electronic medication adherence products as well as the number of task errors.

We examined the usability of electronic medication adherence products by older adults, caregivers, and health care providers. The results of this study are of importance to both older adults and health care providers, especially when determining which electronic medication adherence product can be used to address medication management at home. Higher usability scores indicate that an older adult, caregiver, or health care provider is more likely to be able to use a product appropriately, potentially decrease medication error rates, and improve adherence. Products with lower usability scores may introduce dosing errors, reduce adherence, and, at worst, introduce medication errors. However, although the results of this study demonstrate that some products score well for usability, making them more user-friendly, and others score poorly demonstrating poor usability, there is no benchmark for SUS scores for electronic medication adherence products or for any adherence products for that matter. Therefore, an exact interpretation of this score is difficult. In comparison, the mean SUS scores for other everyday products are as follows: Excel, 56.5; iPhone, 78.5; and microwaves, 86.9 [45,46,48,49]. As a cutoff score above which a product could be deemed highly usable is not available, Bangor et al [45] described an emergence of scoring systems using adjectives such as *acceptable* for products that score above 70 on SUS, *marginal* for scores of 50-69, and *not acceptable* for scores of less than 50. It is easier to relate the product’s usability with the use of these descriptive terms in clinical practice, especially when determining the potential usability of a product. In terms of the products we tested, we would then consider the TimerCap travel size, e-pill MedGlider home medication system, and the eNNOVEA Weekly Planner with Advanced Auto Reminder as products which have *acceptable* usability, whereas the TimerCap universal size, Jones medication adherence system, Reizen vibrating pill box, VitaCarry advanced pill case, MedGlider system 1 with talking reminder, Patterson medical TabTimer super 8, 100-Hour pill reminder, Med-Q smart pill box, MedCentre system, and e-pill multi-alarm pocket XL as *marginally acceptable*, and the remaining products as *unacceptable* for user-friendliness. This type of classification would allow clinicians to evaluate the potential problems with usability their older adult population would encounter. It also allows older adults and caregivers to evaluate products before making a financial investment.

Similar to SUS, the NASA-TLX has been applied in various fields. A meta-analysis of NASA-TLX scores in over 200 publications reported an overall range of scores from 6.21 to 88.5 for various activities (eg, air traffic control, cognitive activities, daily activities, and driving care) [47]. As with the SUS scores, no benchmark for the NASA-TLX regarding medication adherence products, electronic or not, is available. This limits the ability to generalize the scores to a level of workload, that is, to determine the cutoffs for scores that could be used to designate specific products as excellent, moderate, or poor, as has been recommended with SUS scores. Furthermore, adjectives such as those that exist with SUS are not available to allow us to easily apply the scoring in clinical practice. However, the NASA-TLX scores can be compared between the different products to gauge if the workload required to use a product in a population such as ours would be high or not, relative to other products tested. Additionally, by plotting the scores of SUS and NASA-TLX for each product, it became apparent that they were inversely related, that is, products that scored high for usability also scored low for workload. We were also able to demonstrate that the 2 variables are highly correlated (Pearson’s *r*=−0.877; 95% CI −0.907 to 0.839; *df*=184; *P*<.001). Given this finding, it is reasonable to expect that a product that scores well on usability does not place an undue workload cost on the human user.

### Strengths of the Study

Older adults rarely report the problems they have with medication management, and often, they develop their own strategies to address these problems, including the use of commercially available electronic medication adherence products. Our study is the first to compare a number of these products, and the results from our study indicate that usability, workload, time taken to complete tasks, and task completion rates vary between different products. The results of our study provide some evidence for factors to consider when choosing electronic medication adherence products in older adults at risk of medication nonadherence and can also help clinicians and caregivers to guide the choice of an electronic medication adherence product. Although much work needs to be done to investigate the impact of product choice on adherence and clinical outcomes, our study provides evidence to guide the first step in the process, that is, the choice of the product to use.

### Limitations of the Study

This study has some limitations. The participants tested these products in an unfamiliar environment (the University of Waterloo School of Pharmacy) while being observed by research team members, which may have produced or increased anxiety in some of the participants and impacted their ability to use the products efficiently and effectively. Furthermore, participants were asked to test a product they had not been previously exposed to, and this may have impacted the scoring; gaining familiarity with products usually improves the ease of use. Participants were also asked to test 5 products, and frustration with one or more products may have filtered over to the other products they were asked to test thereafter.

### Conclusions

As few studies have investigated the usability of electronic medication adherence products, benchmarks do not yet exist to compare the usability and workload of these products. Our study showed that some electronic medication adherence products may be easier to use than others. Furthermore, information such as usability and workload will provide older adults and clinicians with factors to consider when recommending or purchasing devices to address medication management among older adults.

## Abbreviations

ANOVA: analysis of variance
NASA-TLX: NASA-task load index
SUS: system usability scale

## References

[1] United Nations (2017). World Population Ageing 2017 Highlights.

[2] Suzman R, Beard J (2011). World Health Organization.

[3] Salive ME (2013). Multimorbidity in older adults. Epidemiol Rev.

[4] Vasilopoulos T, Kotwal A, Huisingh-Scheetz MJ, Waite LJ, McClintock MK, Dale W (2014). Comorbidity and chronic conditions in the national social life, health and aging project (NSHAP), wave 2. J Gerontol B Psychol Sci Soc Sci.

[5] Canadian Institute for Health Information (2018). Drug Use Among Seniors in Canada, 2016.

[6] Charlesworth CJ, Smit E, Lee DS, Alramadhan F, Odden MC (2015). Polypharmacy among adults aged 65 years and older in the United States: 1988-2010. J Gerontol A Biol Sci Med Sci.

[7] Payne RA (2016). The epidemiology of polypharmacy. Clin Med (Lond).

[8] Maher RL, Hanlon J, Hajjar ER (2014). Clinical consequences of polypharmacy in elderly. Expert Opin Drug Saf.

[9] Leelakanok N, Holcombe A, Lund B, Gu X, Schweizer M (2017). Association between polypharmacy and death: a systematic review and meta-analysis. J Am Pharm Assoc (2003).

[10] Salvi F, Marchetti A, D'Angelo F, Boemi M, Lattanzio F, Cherubini A (2012). Adverse drug events as a cause of hospitalization in older adults. Drug Saf.

[11] Chiatti C, Bustacchini S, Furneri G, Mantovani L, Cristiani M, Misuraca C, Lattanzio F (2012). The economic burden of inappropriate drug prescribing, lack of adherence and compliance, adverse drug events in older people: a systematic review. Drug Saf.

[12] Butler R, Davis T, Johnson W, Gardner H (2011). Effects of nonadherence with prescription drugs among older adults. Am J Manag Care.

[13] Moser SS, Yu J, Goldshtein I, Ish-Shalom S, Rouach V, Shalev V, Modi A, Chodick G (2016). Cost and consequences of nonadherence with oral bisphosphonate therapy: findings from a real-world data analysis. Ann Pharmacother.

[14] Ip Q, Malone DC, Chong J, Harris RB, Labiner DM (2018). Economic impact of epilepsy and the cost of nonadherence to antiepileptic drugs in older medicare beneficiaries. Epilepsy Behav.

[15] Lloyd JT, Maresh S, Powers CA, Shrank WH, Alley DE (2019). How much does medication nonadherence cost the medicare fee-for-service program?. Med Care.

[16] World Health Organization (2013). Adherence to Long-Term Therapies: Evidence for Action.

[17] Wroe AL (2002). Intentional and unintentional nonadherence: a study of decision making. J Behav Med.

[18] Mukhtar O, Weinman J, Jackson SH (2014). Intentional non-adherence to medications by older adults. Drugs Aging.

[19] Yap AF, Thirumoorthy T, Kwan YH (2016). Systematic review of the barriers affecting medication adherence in older adults. Geriatr Gerontol Int.

[20] Mullen R, Curtis L, O'Conor R, Serper M, McCarthy D, Bailey S, Parker R, Wolf M (2018). Visual acuity, literacy, and unintentional misuse of nonprescription medications. Am J Health Syst Pharm.

[21] Philbert D, Notenboom K, Bouvy ML, van Geffen EC (2014). Problems experienced by older people when opening medicine packaging. Int J Pharm Pract.

[22] Sino CG, Sietzema M, Egberts TC, Schuurmans MJ (2014). Medication management capacity in relation to cognition and self-management skills in older people on polypharmacy. J Nutr Health Aging.

[23] Notenboom K, Beers E, van Riet-Nales DA, Egberts TC, Leufkens HG, Jansen PA, Bouvy ML (2014). Practical problems with medication use that older people experience: a qualitative study. J Am Geriatr Soc.

[24] Atkin PA, Finnegan TP, Ogle SJ, Shenfield GM (1994). Functional ability of patients to manage medication packaging: a survey of geriatric inpatients. Age Ageing.

[25] Spiers M, Kutzik D, Lamar M (2004). Variation in medication understanding among the elderly. Am J Health Syst Pharm.

[26] Cárdenas-Valladolid J, Martín-Madrazo C, Salinero-Fort MA, de-Santa Pau EC, Abánades-Herranz JC, de Burgos-Lunar C, PATER Group (2010). Prevalence of adherence to treatment in homebound elderly people in primary health care: a descriptive, cross-sectional, multicentre study. Drugs Aging.

[27] Maddigan SL, Farris KB, Keating N, Wiens CA, Johnson JA (2003). Predictors of older adults' capacity for medication management in a self-medication program: a retrospective chart review. J Aging Health.

[28] Farris KB, Phillips BB (2008). Instruments assessing capacity to manage medications. Ann Pharmacother.

[29] Elliott RA, Marriott JL (2009). Standardised assessment of patients' capacity to manage medications: a systematic review of published instruments. BMC Geriatr.

[30] Gellad WF, Grenard JL, Marcum ZA (2011). A systematic review of barriers to medication adherence in the elderly: looking beyond cost and regimen complexity. Am J Geriatr Pharmacother.

[31] Cross A, Elliott R, George J (2016). Interventions for improving medication-taking ability and adherence in older adults prescribed multiple medications. Cochrane Database Syst Rev.

[32] Costa E, Giardini A, Savin M, Menditto E, Lehane E, Laosa O, Pecorelli S, Monaco A, Marengoni A (2015). Interventional tools to improve medication adherence: review of literature. Patient Prefer Adherence.

[33] Farooqi M, Carter C, Patel T (2017). Electronic medication adherence technologies’ classification to guide use in older adults: Pharmacy practice research abstracts: Canadian Pharmacists Conference 2017. Can Pharm J.

[34] Zapata BC, Fernández-Alemán JL, Idri A, Toval A (2015). Empirical studies on usability of mhealth apps: a systematic literature review. J Med Syst.

[35] Daniels J, Fels S, Kushniruk A, Lim J, Ansermino JM (2007). A framework for evaluating usability of clinical monitoring technology. J Clin Monit Comput.

[36] Wildenbos G, Peute L, Jaspers M (2015). A framework for evaluating mhealth tools for older patients on usability. Stud Health Technol Inform.

[37] Reeder B, Demiris G, Marek KD (2013). Older adults' satisfaction with a medication dispensing device in home care. Inform Health Soc Care.

[38] Tseng M, Wu H (2014). A cloud medication safety support system using QR code and web services for elderly outpatients. Technol Health Care.

[39] Ligons FM, Romagnoli KM, Browell S, Hochheiser HS, Handler SM (2011). Assessing the usability of a telemedicine-based medication delivery unit for older adults through inspection methods. AMIA Annu Symp Proc.

[40] Ligons FM, Mello-Thoms C, Handler SM, Romagnoli KM, Hochheiser H (2014). Assessing the impact of cognitive impairment on the usability of an electronic medication delivery unit in an assisted living population. Int J Med Inform.

[41] Grindrod KA, Li M, Gates A (2014). Evaluating user perceptions of mobile medication management applications with older adults: a usability study. JMIR Mhealth Uhealth.

[42] Stuck RE, Chong AW, Mitzner TL, Rogers WA (2017). Medication management apps: usable by older adults?. Proc Hum Factors Ergon Soc Annu Meet.

[43] Jaspers MWM (2009). A comparison of usability methods for testing interactive health technologies: methodological aspects and empirical evidence. Int J Med Inform.

[44] Brook J, Jordan PW, Thomas B, McClelland IL, Weerdmeester B (1996). SUS-a quick and dirty usability scale. Usability Evaluation in Industry.

[45] Bangor A, Staff T, Kortum P, Miller J, Staff T (2009). Determining what individual SUS scores mean: adding an adjective rating scale. J Usability Stud.

[46] Hart SG, Staveland LE (1988). Development of NASA-TLX (task load ondex): results of empirical and theoretical approach. Adv Psychol.

[47] Grier RA (2015). How high is high? A meta-analysis of NASA-TLX global workload scores. Proc Hum Factors Ergon Soc.

[48] Kushniruk AW, Patel VL (2004). Cognitive and usability engineering methods for the evaluation of clinical information systems. J Biomed Inform.

[49] Sauro J, Lewis J (2016). Quantifying the User Experience: Practical Statistics for User Research. Second Edition.

